# Effects of SARS-CoV-2 infection on embryological outcomes in assisted reproductive technology during the Omicron epidemic

**DOI:** 10.1186/s13048-023-01301-3

**Published:** 2023-11-22

**Authors:** Yuling Mao, Yachao Yao, Hongzi Du, Zheng Wang, Xueliang Zhou, Ming Zeng, Chunyan Wang, Hanyan Liu, Yang Luo, Honghai Hong, Jianqiao Liu, Lei Li

**Affiliations:** 1 Department of Obstetrics and Gynecology, Center for Reproductive Medicine, Guangdong Provincial Key Laboratory of Major Obstetric Diseases, Guangdong Provincial Clinical Research Center for Obstetrics and Gynecology, Guangdong-Hong Kong-Macao Greater Bay Area Higher Education Joint Laboratory of Maternal-Fetal Medicine, The Third Affiliated Hospital of Guangzhou Medical University, Guangzhou, China; 2 Key Laboratory for Reproductive Medicine of Guangdong Province, The Third Affiliated Hospital of Guangzhou Medical University, Guangzhou, China; 3 Department of Laboratory medicine, Guangdong Second Provincial General Hospital, Guangzhou, China; 4 Department of Clinical Laboratory, The Third Affiliated Hospital of Guangzhou Medical University, Guangzhou, China

**Keywords:** SARS-CoV-2, Follicular fluid, Immature oocyte, Granular cell, Blastocyst, Embryological outcome

## Abstract

**Background:**

The influence of severe acute respiratory syndrome coronavirus 2 (SARS-CoV-2) infection on assisted reproductive technology (ART) has received increasing attention. It has been reported that the SARS-CoV-2 RiboNucleic Acid (RNA) cannot be detected in follicular fluid and granulosa cells. However, the detection rate of SARS-CoV-2 RNA in immature oocytes and blastocysts has still unknown. Moreover, the effect of SARS-CoV-2 infection on embryological outcomes in ART during the Omicron epidemic is limited.

**Methods:**

A prospective study was performed to explore the detection rate of viral RNA in biological specimens from patients who tested positive for SARS-CoV-2 RNA and the effects of SARS-CoV-2 infection on embryological outcomes. A total of 211 patients underwent transvaginal oocyte retrieval at the Third Affiliated Hospital of Guangzhou Medical University between December 13, 2022 and December 30, 2022. Prior to transvaginal oocyte retrieval, 61 individuals tested positive for SARS-CoV-2 RNA within 24 h. Follicular fluid was preserved during oocyte retrieval. Granular cells were collected after degranulation (Intracytoplasmic sperm injection only). Immature oocytes were collected at the end of the ICSI. Unavailable blastocysts were collected on day 6 (D6). The TIANLONG SARS-CoV-2 RT-PCR-Kit was used to detect SARS-CoV-2 RNA in all samples. The COVID-19 and Non COVID-19 groups were contrasted in the following areas: fertilization rate, 2PN rate, Day 3 (D3) available embryos rate, D3 good-quality embryos rate, blastocyst formation rate, good-quality blastocyst formation rate.

**Results:**

All samples were negative except for an immature oocytes sample that was positive for SARS-CoV-2 viral RNA with a detection rate of 6.67%. Whether in-vitro fertilization (IVF) or intracytoplasmic sperm injection (ICSI), the rate of fertilization, 2PN, D3 available embryos, D3 good-quality embryos, blastocyst formation, good-quality blastocyst formation was not significantly negative different between the COVID-19 and the Non COVID-19 groups. Our findings were validated by an overview of the embryological outcome from the cycles before SARS- Cov-2 infection from the same patient.

**Conclusions:**

Except for immature oocytes, none of the follicular fluid, granulosa cells, or blastocysts samples contained viral RNA. In addition, SARS-CoV-2 infection had no detrimental effects on the embryological outcomes of ART.

## Introduction

Coronavirus disease-2019 (COVID-19), which was initially identified in China on December 30, 2019, is caused by the novel severe acute respiratory syndrome coronavirus 2 (SARS-CoV-2) [1]. Since then, SARS-CoV-2 has spread across the globe, resulting in millions of infected patients and deaths worldwide [2, 3]. Due to the alarming rate of community transmission and relatively high mortality rates associated with COVID-19, this flu pandemic resulted in an unparalleled global health crisis. Thus, social and traveling restrictions were implemented in most countries, including the suspension of infertility treatment services.

With the relaxation of social distancing measures in many countries, there was a gradual resumption of ART services. Thus, new scientific questions about the effects of viral infections on assisted reproductive technology (ART) outcomes were raised. Various guidelines for fertility treatments have been proposed [4–6], whereas other studies have focused on discovering a connection between ART and COVID-19 at the cellular level of gametes and embryos [7, 8].

In broad terms, the SARS-CoV-2 is believed to affect mainly the respiratory system [9]. Currently, SARS-CoV-2 infection in the female reproductive system is still uncertain and controversial. The majority of researches have shown that SARS-CoV-2 cannot be found in follicular fluid [10, 11] or granulosa cells [12]. However, the risk of SARS-CoV-2 in oocytes [13] and embryos during ART is unknown due to the paucity of available evidence. Therefore, our study aimed to detect SARS-CoV-2 RNA in follicular fluid, granulosa cells, oocytes and embryos. And to explore the possibility of virus transmission during the operation process in the assisted reproductive embryo laboratory as well as the potential effects of COVID-19 infection on vertical transmission.

Most studies have not found a significant effect of COVID-19 on laboratory outcomes. A study showing the effect of SAR-CoV-2 infection on women’s fertility was published in July 2021 by Wang et al. [14] Only the rate of blastocyst formation was noticeably lower when they compared the number of retrieved oocytes, mature oocyte rate, fertilization rate, and number of retrieved oocytes. Nine couples’ IVF results were compared before and after COVID-19 infection by Orvieto et al. [15]. The number of oocytes obtained and the rate of fertilization were similar; however, the proportion of top-quality embryos (TQE) was significantly lower. However, most studies have focused on the original, α, β, and γ variants, most of which are past infections. The possible effects of Omicron variants (discovered in November 2021) on embryological outcome are largely unknown. Omicron was the main strain in circulation after China’s epidemic prevention policy was relaxed. At present, no direct evidence indicates that SARS-CoV-2 negatively influences human reproduction [16], and whether SARS-CoV-2 affects gametes and embryonic development remains to be clarified. We aimed to examine the presence of viral RNA in biological samples from positive patients with SARS-CoV-2. We also aimed to evaluate whether there was a measurable effect of Omicron variants on the embryological outcome of ART and provide a theoretical framework for routine ART procedures.

## Methods

### Study design and patients

All women scheduled for transvaginal oocyte retrieval for ART were asked to undergo nasopharyngeal swab screening. In case of a positive Reverse Transcription-Polymerase Chain Reaction (RT-PCR) test less than 24 h before oocyte retrieval, the patient or couple was given advice regarding the potential effect of viral infection on the outcome of ART treatment as well as the unknown risk of vertical transmission. If the patient or couple choose to proceed, the procedure was performed as scheduled. Following all necessary protective safety measures according to the best practice guidelines, transvaginal oocyte retrieval was carried out in a dedicated operating room, with all the medical staffs in third-level protection and patients wearing N95 masks.

If the male partner was positive, oocytes were collected, granulosa cells were removed, and oocytes were frozen in an adjacent temporary embryo laboratory. If the male PCR result was negative, the oocytes were transported to a regular embryo laboratory for insemination in an insulated closed box. Oocytes and gametes from positive patients with positive RT-PCR tests were cultured in separate incubators, set on a Cryotop strip (Kitazato Corp., Japan), and stored in a special liquid nitrogen tank (Taylor Wharton HC35, Theodore, AL, USA).

Between December 13, 2022 and December 30, 2022, 211 patients underwent oocyte retrieval at the Third Affiliated Hospital of Guangzhou Medical University; of these, 28.91% (61/211) tested positive for SARS-CoV-2 < 24 h before oocyte retrieval.

The patients were classified into two groups: non COVID-19 patients (n = 150), who tested negative for COVID-19 < 24 h before oocyte retrieval, and COVID-19 patients (n = 61), who tested positive for COVID-19 < 24 h before oocyte retrieval. The study was approved by the Ethics Committee at the Third Affiliated Hospital of Guangzhou Medical University, and written informed consent was obtained from each participant.

### Follicular fluid collection

The follicular fluid was preserved during oocyte retrieval. Only macroscopically clear fluids, indicating lack of contamination and blood, were considered in the study. Follicular fluid from the first punctured follicle from each ovary was discarded to minimize the risk of contamination with vaginal mucus and cells, except if only one follicle had been punctured. Then 1 ml was transferred into sterile RNAse-free sample preservation tubes immediately. And then the samples were stored at 4 °C for SARS-CoV-2 RT-PCR tests at a later stage.

### Granulosa cells

Degranulation was carried out in accordance with the embryo laboratory standard operating procedure. For ICSI and oocyte vitrification, 15 granulosa cells which were obtained from patients with immature oocyte were transferred to sterile RNAse-free sample preservation tubes and stored at 4 °C for SARS-CoV-2 RT-PCR test after oocyte denudation. However, 4 granulosa cell samples could not be amplified by PCR, only 11 granulosa cell results were shown in this paper. Among the 11 granulosa cell samples, two were obtained from ICSI and nine from oocytes vitrification. For IVF insemination, granulosa cells were denuded the day after insemination, but no granulosa cells were collected for the analysis.

### Immature oocyte

After ICSI and oocyte vitrification, immature oocytes were collected and transferred to sterile RNAse-free sample preservation tubes and stored at 4 °C for SARS-CoV-2 RT-PCR. Immature oocytes from the same patient were all packed into the same tube and not stored separately.

### Blastocysts

Depending on the patient’s age and other conditions, one or two embryos were vitrified on D3, and the remaining embryos continued the culturing process. Available blastocysts formed on days 5 and 6 were also vitrified. The unavailable blastocysts (grade 1 blastocyst, grade 2 blastocyst, 3CC, 4CC, 5CC, 6CC according to Gardner’s score) on day 6 from different patients were collected as a sample of the embryo. Once transferred to sterile RNAse-free sample preservation tubes, samples were stored at 4 °C until assayed.

### SARS-CoV-2 detection

All SARS-CoV-2 samples were analyzed at our hospital. RNA was extracted from 211 nasopharyngeal swab samples, 61 follicular fluid, 11 granulosa cells samples, 15 immature oocytes samples and 3 blastocyst samples using the nucleic acid extraction or purification kit (TIANLONG, #T183, China) according to the manufacturer’s protocol. Molecular detection using RT-PCR was performed using the SARS-CoV-2 RT-PCR Kit to detect SARS-CoV-2–specific RNA (TIANLONG, #ZC-HX-201-2, China) according to the manufacturers’ instructions. This kit uses real-time fluorescent PCR technology to design specific primers and TaqMan probes for the ORF1ab and N genes of novel coronavirus 2019-nCoV, amplified using a fluorescent quantitative PCR instrument (SLAN-96P) to detect nucleic acid of the novel coronavirus 2019-nCoV. Endogenous RNase P was set as the internal reference. Cycle threshold value of < 40 was considered positive [17].

### Outcome measure

The primary outcomes of this study included virus detection rate, oocyte maturity rate, fertilization rate, 2PN rate, D3 available embryos rate, D3 good-quality embryos rate, blastocyst formation rate and good-quality blastocyst formation rate. The virus detection rate in the sample was measured by dividing the number of positive cases by the total number of tests. The fertilization rate was calculated as the number of fertilized oocytes divided by number of metaphase II oocytes in ICSI cases and the total number of oocytes in IVF case. Normal fertilization rate was defined as the proportion of 2PN fertilization to the total number of oocytes (IVF) or injected oocytes (ICSI), respectively. D3 available embryos rate refers to the number of available embryos (those with blastomere numbers more than four, fragmentation ≤ 20%, and without obvious size differences in blastomeres.) on the third day divided by the total number of cleavage embryos. The D3 good-quality embryos rate refers to the number of high-quality embryos on the third day divided by 2PN cleavage embryos.

Good-quality cleavage-stage embryos were defined as those with 7–9 symmetrical blastomeres without obvious fragmentation [18]. Good-quality blastocysts were defined as those that reached at least grade 3 expansion and grade A or B for the inner-cell mass and trophectoderm parameters [19].

### Statistical analysis

The statistical analysis was performed using the Statistical Package for Social Science (SPSS) version 22.0. The baseline characteristic was expressed as the mean ± SD (standard deviation) and differences in variables were compared using Student’s t-test. Categorical variables were described as frequencies and percentages, and compared using chi-square test. *P* < 0.05 was considered statistically significant.

## Results

### Study population

The characteristics of the study population are shown in Table 1. Between December 13, 2022 and December 30, 2022, 211 patients underwent oocyte retrieval at the Third Affiliated Hospital of Guangzhou Medical University; of these, 28.91% (61/211) tested positive for SARS-CoV-2 within 24 h before oocyte retrieval. The treatment outcome depends on the nucleic acid results of the male within 24 h before oocyte retrieval. If the male partners tested positive for SARS-CoV-2, oocyte vitrification is performed (n = 65); 146 patients underwent IVF or ICSI with partner negative for the nasopharyngeal SARS-CoV-2 test. Twenty-three women in the COVID-19 insemination group and 123 women in the non-COVID-19 insemination group were included.

**Table 1:** Pharyngeal swab nucleic acid results and treatment outcomes of patients

	Treatment outcome	Number of cycles	%
Female positive and male positive	oocyte vitrification	38	18.01
Female positive and male negative	insemination	23	10.90
Female negative and male positive	oocyte vitrification	27	12.80
Female negative and male negative	insemination	123	58.29
total		211	100

### Detection of viral RNA in biological specimens from SARS-CoV-2 patients

We have conducted a prospective study on residual material during ART treatment in women positive for SARS-CoV-2. SARS-CoV-2 viral RNA was determined in follicular fluid, granulosa cells, immature oocytes and blastocysts from women positive for SARS-CoV-2 (Table 2). The results revealed that 6.67% (1/15) of the immature oocyte from patients with COVID-19 were positive for SARS-CoV-2 viral RNA, whereas viral RNA was not detected in any of the follicular fluid, granulosa cells and blastocysts samples.

**Table 2:** PCR outcome of biological specimens from women positive for SARS-CoV-2

	Number of cycles	Number of positives	Positive rate (%)
Follicular fluid	61	0	0
Granulosa cells	11	0	0
Immature oocytes	15	1	6.67
Discarded blastocysts	3	0	0

PCR, Polymerase Chain Reaction; SARS-CoV-2, severe acute respiratory syndrome coronavirus 2

### Cycle characteristics of patients who underwent insemination

The characteristics of the patients who underwent insemination are presented in Table 3. No significant differences were discovered in the overall patient age (range: 22–45 years). Patients in the COVID-19 group and the non-COVID-19 group had similar BMI and infertility duration in IVF and ICSI. Similarly, there were no differences between the two groups regarding the reasons for infertility in the IVF group. However, in the ICSI group, the proportion of male infertility was significantly higher in the COVID-19 group, and the difference was statistically significant.

**Table 3:** Cycle characteristics of patients who underwent insemination

	IVF			ICSI		
	COVID-19 (n = 13)	Non COVID-19 (n = 64)	*P* value	COVID-19 (n = 10)	Non COVID-19 (n = 59)	*P* value
Age (years)	35.05 ± 5.26	33.22 ± 5.11	0.068	34.86 ± 5.37	33.35 ± 5.13	0.744
BMI (kg/m ^2^)	21.78 ± 2.86	21.66 ± 2.82	0.113	21.52 ± 2.89	21.62 ± 2.80	0.225
Infertility duration (years)	4.97 ± 4.24	4.32 ± 3.83	0.188	5.16 ± 4.25	4.39 ± 3.85	0.581
Type of infertility			0.464			0.522
Primary infertility	3 (23.08%)	23 (35.94%)		7 (70.00%)	35 (59.32%)	
Secondary infertility	9 (69.23%)	41 (64.06%)		3 (30.00%)	24 (40.68%)	
Reasons of infertility			0.699			**0.000**
Female factor	7 (53.85%)	34 (53.13)		1 (10.00%)	26 (44.07%)	
Male factor	2 (15.38%)	6 (9.38%)		5 (50.00%)	18 (30.51%)	
Multiple factors	2 (15.38%)	18 (28.13)		0	13 (22.03)	
Unexplained infertility	2 (15.38%)	6 (9.38%)		4 (40.00%)	2 (3.39%)	

COVID-19, Coronavirus disease-2019; IVF, in-vitro fertilization; ICSI, intracytoplasmic sperm injection; BMI, Body Mass Index

### Embryological outcomes

Table 4 presents the embryological outcomes in patients who underwent IVF insemination. The fertilization rate (72.48%), 2PN rate (51.01%), D3 available embryos rate (63.64%), blastocyst formation rate (35.82%) and good-quality blastocyst formation rate (22.39%) in the COVID-19 group were similar to those in the non-COVID-9 group (*P* > 0.05). The D3 good-quality embryos rate was statistically higher in COVID-19 group compared with non-COVID-19 group (P < 0.05).

**Table 4:** Embryological outcomes of IVF cycles

	COVID-19 (n = 13)	Non COVID-19 (n = 64)	*X* ^*2*^	*P* value	
Fertilization rate (%)	72.48(108/149)	73.53 (425/578)	0.066	0.797	
2PN rate (%)	51.01 (76/149)	46.71 (270/578)	0.876	0.349	
D3 available embryos rate (%)	63.64 (63/99)	53.44 (225/421)	3.370	0.066	
D3 good-quality embryos rate (%)	40.54 (30/74)	26.69 (71/266)	5.317	**0.021**	
Blastocyst formation rate (%)	35.82 (24/67)	36.78 (89/242)	0.021	0.886	
Good-quality blastocyst formation rate (%)	22.39 (15/67)	23.97 (58/242)	0.072	0.788	

IVF, in-vitro fertilization; COVID-19, Coronavirus disease-2019; 2PN, 2 pronucleus; D6, day 6

The embryology data of patients who underwent ICSI insemination are presented in Table 5. Oocyte maturity rate (65.79% vs. 74.29%), fertilization rate (74.66% vs. 80.80%), 2PN rate (65.33% vs. 71.74%), D3 available embryos rate (42.86% vs. 50.45%), D3 good-quality embryos rate (16.33% vs. 24.81%), blastocyst formation rate (35.48% vs. 45.77%), and good-quality blastocyst formation rate (12.90% vs. 22.57%) revealed a decreasing trend; however, there was no statistical difference in the results.

**Table 5:** Embryological outcomes of ICSI cycles

	COVID-19 (n = 10)	Non COVID-19 (n = 59)	*X* ^*2*^	*P* value	
Oocyte maturity rate (%)	65.79 (75/114)	74.29 (552/743)	3.640	0.056	
Fertilization rate (%)	74.66 (56/75)	80.8 (446/552)	1.555	0.212	
2PN rate (%)	65.33 (49/75)	71.74 (396/552)	1.315	0.251	
D3 available embryos rate (%)	42.86 (24/56)	50.45 (224/444)	1.147	0.284	
D3 good-quality embryos rate (%)	16.33 (8/49)	24.81 (98/395)	1.726	0.189	
Blastocyst formation rate (%)	35.48 (11/31)	45.77 (146/319)	1.208	0.272	
Good-quality blastocyst formation rate (%)	12.9 (4/31)	22.57 (72/319)	1.553	0.213	

ICSI, intracytoplasmic sperm injection; COVID-19, Coronavirus disease-2019; 2PN, 2 pronucleus; D3, day 3

### Comparison with the preceding ART cycle

To further evaluate the impact of SARS-CoV-2 infection on embryo development, we present the descriptive embryology data of the ART cycle during SARS-CoV-2 infection with those in the previous cycle in the same patient (Table 6). Seven patients had at least one previous IVF/ICSI cycle in our center (Table 6). Among the seven patients, only patients 3,5 and 7 had no changes in protocols for ovarian stimulation and fertilization method. Oocyte retrieval for these three patients was less than half a year; thus, their data were comparable. All cycles of the three patients were IVF, and the normal fertilization rate in the acute COVID-19 infection group was higher than that in the group without infection, whereas the blastocyst formation rate was decreased in all patients. The trends of total fertilization rate, D3 available embryo rate and D3 good embryo rate were inconsistent. In patients 1,2,4, and 6, the interval between different cycles was long (6–60 months), and there were changes in ovarian stimulation protocols and fertilization method, all of which had definite effects on embryonic development; therefore, a comparative analysis was not possible.

**Table 6:** Overview of the embryological outcome from the cycles before SARS-CoV-2 infection from the same patient

Patient	cycle number	SARS-CoV-2 infection	protocols for ovarian stimulation	Fertilization method	Fertilization rate (%)	Oocyte maturity rate (%)	2PN rate (%)	D3 available embryos rate (%)	D3 good-quality embryos rate (%)	Blastocyst formation rate (%)	Good-quality blastocyst formation rate (%)	
1	1	No	Ultra-long	IVF	66.67% (4/6)	N/A	16.67% (1/6)	0 (0/1)	0 (0/1)	N/A	N/A	
1	2	No	Long-acting long	IVF	0%(0/16)	N/A	N/A	N/A	N/A	N/A	N/A	
1	3	No	Antagonist	ICSI	100%(3/3)	100% (3/3)	100% (3/3)	66.67% (2/3)	33.33% (1/3)	N/A	N/A	
1	4	Yes	Mini-stimulation	ICSI	66.67% (2/3)	37.5% (3/8)	66.67% (2/3)	66.67% (2/3)	50% (1/2)	N/A	N/A	
2	1	No	Long-acting long	IVF	77.27% (17/22)	N/A	63.64% (14/22)	82.35% (14/17)	50% (7/14)	50% (7/14)	35.71% (5/14)	
2	2	Yes	Antagonist	IVF	83.33% (10/12)	N/A	33.33% (4/12)	50% (5/10)	25% (1/4)	0 (0/8)	0 (0/8)	
3	1	No	Antagonist	IVF	33.33% (5/15)	N/A	26.67% (4/15)	80% (4/5)	25% (1/4)	33.33% (1/3)	33.33% (1/3)	
3	2	Yes	Antagonist	IVF	**68.42% (13/19)** ^*^	N/A	57.89% (11/19)	53.85% (7/13)	18.18% (2/11)	31.5% (3/8)	12.5% (1/8)	
4	1	No	Antagonist	IVF	70% (7/10)	N/A	60% (6/10)	42.86% (3/7)	50% (3/6)	50% (3/6)	33.33% (2/6)	
4	2	No	Long-acting long	IVF	46.15% (6/13)	N/A	30.77% (4/13)	16.67% (1/6)	0 (0/4)	0 (0/3)	0 (0/3)	
4	3	Yes	Antagonist	ICSI	60% (3/5)	100% (5/5)	40% (2/5)	33.33% (1/3)	0 (0/2)	50% (1/2)	0 (0/2)	
5	1	No	Antagonist	IVF	50% (7/14)	N/A	28.57% (4/14)	42.86% (3/7)	0 (0/4)	50% (1/2)	50% (1/2)	
5	2	Yes	Antagonist	IVF	**90% (9/10)** ^*^	N/A	**90% (9/10)** ^*^	**88.89% (8/9)** ^*^	**66.67% (6/9)** ^*^	42.86% (3/7)	28.57% (2/7)	
6	1	No	Antagonist	ICSI	66.67% (6/9)	75% (9/12)	66.67% (6/9)	100% (6/6)	50% (3/6)	33.33% (2/6)	16.67% (1/6)	
6	2	Yes	Long-acting long	ICSI	44.44% (4/9)	81.82% (9/11)	44.44% (4/9)	50% (2/4)	25% (1/4)	50% (2/4)	25% (1/4)	
7	1	No	Mini-stimulation	IVF	100% (1/1)	N/A	0 (0/1)	0 (0/1)	− (0/0)	0(0/1)	0 (0/1)	
7	2	Yes	Mini-stimulation	IVF	50% (2/4)	N/A	50% (2/4)	50% (1/2)	50% (1/2)	0(0/1)	0 (0/1)	

SARS-CoV-2, severe acute respiratory syndrome coronavirus 2; 2PN, 2 pronucleus; D3, day 3; IVF, in-vitro fertilization; ICSI, intracytoplasmic sperm injection

*The embryological outcome of the same patient was statistically different in different cycles

## Discussion

In this large prospective cohort study of patients that underwent ART treatment from December 13, 2022 to December 30, 2022, there were no positive SARS-CoV-2 RT-qPCR results among follicular fluid and granulosa cells samples collected for analysis. The data presented in this study demonstrate for the first time that the undetectable of SARS-CoV-2 RNA in discarded blastocysts and the presence in immature oocytes of patients with COVID-19 who underwent ART treatments; the detection rate was 6.67%. Finally, acute SARS-CoV-2 infection had no adverse impact on embryological outcomes.

Since the initial identification of COVID-19 in Wuhan province, China, in late 2019 [20], many studies have focused on the impact of SARS-CoV-2 infection on the lungs; however, little is known about whether this virus affects human reproductive health. SARS-CoV-2 was not found in vaginal swab samples from 10 women who had been diagnosed with COVID-19 [21]. In 35 female patients with severe COVID-19 patients who were in the postpartum and postmenopausal stage, COVID-19 was not found in vaginal fluid or exfoliated cells [22]. In Six of 38 patients with COVID- 19, SARS-CoV-2 was detected in semen [23]; one of 12 COVID-19 patients tested SARS-CoV-2-positive during postmortem examination [24]. In pregnant women, SARS-CoV-2 was also localized predominantly in syncytiotrophoblast cells [25], amniotic and placental [26].

The infection of the cells involves the presence of Zinc metallopeptidase angiotensin-converting enzyme 2 (ACE2) receptors and priming and cleavage of the virus spike proteins by transmembrane protease serines 2 (TMPRSS2) [27, 28]. In theory, cells expressing high levels of ACE2 may be highly susceptible to viral entry. ACE2 is expressed in many systems [29]. Evidence suggests that SARS-CoV-2 infects male urogenital tract tissues [30]. In males ACE2 expression is relatively high in testis and the corresponding protein is also present in the mature spermatozoa, Sertoli cells and Leydig cells, and consistently the virus has been detected in semen samples [31–33]. ACE2 is also highly expressed in the uterus, ovaries, and placenta [34]. These observations suggested that viral infection could potentially compromise fertility status. Moreover, since ACE2 and TMPRSS2 virus receptors were identified in reproductive organs [28], it became necessary to evaluate the risk of biological specimens for contamination with SARS-CoV-2 in patients undergoing ART cycles.

The presence of ACE2 on the follicle surface [35] has been investigated recently. However, some investigations assessed the viral RNA levels in the follicular fluid from COVID-19 infected women [10–12]. Our data confirm these reports that acute infection with SARS-CoV-2 did not contaminate follicular fluid in infected women during the Omicron epidemic. In contrast, ACE2 and TMPRSS2 were not co-expressed in non-human and human granulosa cells [36], implying that granulosa cells may be a physical barrier to SARS-CoV-2 infection. Consistent with these conclusions, none of the women included in the study had positive SARS-CoV-2 results in granulosa cells. These results confirm the possible risk of contamination in ART laboratories.

Based on available data, co-expression of TMPRSS2 and ACE2 was also observed in oocytes [10, 35]. Therefore, it could be possible that SARS-CoV-2 may target oocytes. However, there is limited data concerning COVID-19 and oocytes. In one study, 16 oocytes from two women who are positive for SARS-CoV-2 were tested for viral mRNA, and all samples were negative [13]. Contrary to this report, we expanded the sample size and found that 6.67% (1/15) of the immature oocyte from patients with COVID-19 were positive for SARS-CoV-2 viral RNA. The PCR results of this sample revealed a CT value of 36 for the O gene and 37 for the N gene. We confirmed the presence of SARS-CoV-2 RNA in oocytes for the first time in this study. This result suggests that gametes and zygotes from patients positive for COVID-19 should be cultured and frozen independently. However, because several immature oocytes were mixed in a test tube, it was impossible to know whether the virus was present in each immature oocyte.

Currently, few studies have focused on the presence of the SARS-CoV-2 in embryos. ACE2 is highly expressed in human germ cells and pre-implantation embryos [37]. The co-expression of TMPRSS2 and ACE2 is highest on Day 6 in trophectoderm (TE) cells [38], suggesting that TE cells may be particularly vulnerable to SARS- CoV-2 during that time. This implicates that SARS-CoV-2 may bind to and infect embryos. However, we collected discarded blastocysts from three patients positive for COVID-19, none of which tested positive for SARS-CoV-2 RNA. To our knowledge, this is the first report on the absence of viral RNA in blastocysts of patients with SARS-CoV-2. However, due to the limited sample size, the findings should be interpreted with caution.

With the relaxation of social distancing measures, ART services gradually resumed. Thus, new scientific questions about the effects of COVID-19 on ART outcomes were raised. When comparing treatment of the same couples before and after SARS-CoV-2 infection, there was no difference in embryological variables (number of oocytes and mature oocytes, fertilization rate), according to a small case series with nine couples [15]. In a retrospective study [39], a group from Wuhan examined patients for IVF during a 10-month interval and compared 65 women seropositive (IgM or IgG) for SARS-CoV-2 to 195 controls; laboratory and clinical outcomes were similar in both groups. Retrospective comparisons were made between the results of the first fresh ART cycle in 121 patients with prior COVID-19 (within 12 months of diagnosis) and 121 patients without prior COVID-19 [40]. Previous SARS-CoV-2 infection was discovered to have no impact on oocyte yield, maturation rate, fertilization rate, number of vitrified embryos and clinical pregnancy rates. However, most studies have focused on past infections and are retrospective studies. In our study, we conducted a prospective study with a large sample size on embryonic outcomes in patients with acute COVID-19 infection during the Omicron epidemic. Our data were reassuring; thus, we suggest that SARS-CoV-2 infection probably may not substantial impact the embryological outcomes of ART.

Further analysis of the embryological outcome from the cycles before SARS-CoV-2 infection in the same patient was performed to assess for the possible effects of acute infection on embryonic development. Similar conclusions were obtained, acute SARS-CoV-2 infection had no negative effect on embryological outcomes.

Based on our results, we believe that it is relatively safe to undergo the ART cycle after infection with COVID-19, and there are no adverse effect on embryonic development. However, since viral RNA has been discovered in oocytes, it is necessary to culture and preserve it separately when operating in the embryo laboratory.

This is the first investigation, as far as we are aware, into the presence or absence of SARS-CoV-2 RNA in immature oocytes and blastocysts. Additionally, this is the largest study to assess the effect of acute SARS- CoV-2 infection on embryonic outcomes in ART cycles.

However, this study had two limitations. First, the number of blastocysts was limited (3 samples). Second, due to the limited follow-up period, none of these patients underwent transplantation; thus, pregnancy and neonatal outcomes are unavailable in this study, which will be further evaluated with an extended follow-up period.

## Conclusions

In conclusion, we suggest that SARS-CoV-2 contamination of follicular fluid, granulosa cells, and blastocysts does not occur in women infected with SARS-CoV-2 undergoing ART. However, a percentage of the virus was detected in immature oocytes. This observational cohort provides compelling evidence for the implementation of additional safety measures by IVF laboratory staff to avoid cross-contamination. Our data suggest that acute SARS-CoV-2 infection does not effect embryological outcomes; however, informed consent is necessary.

## Data Availability

The data sets in this study cannot be publicly available because of involving the patient privacy but are available from the corresponding author on reasonable request.

## List of abbreviations

COVID-19: Coronavirus disease-2019
ART: assisted reproductive technology
SARS-CoV-2: severe acute respiratory syndrome coronavirus 2
TQE: top-quality embryos
SD: standard deviation
ACE2: angiotensin-converting enzyme 2
TE: trophectoderm
Acid RNA: RiboNucleic
D6: day 6
IVF: in-vitro fertilization
ICSI: intracytoplasmic sperm injection
RT-PCR: Reverse Transcription-Polymerase Chain Reaction
TMPRSS2: transmembrane protease serines 2
